# Removal of Acetaminophen from Aqueous Solutions in an Adsorption Process

**DOI:** 10.3390/ma17020431

**Published:** 2024-01-16

**Authors:** Agata Skwarczynska-Wojsa, Alicja Puszkarewicz

**Affiliations:** Department of Water Purification and Protection, Rzeszow University of Technology, Al. Powstancow Warszawy 12, 35-959 Rzeszow, Poland; apuszkar@prz.edu.pl

**Keywords:** adsorption, activated carbon, acetaminophen, paracetamol, kinetics, equilibrium

## Abstract

Acetaminophen (C_8_H_9_NO_2_, also called paracetamol) is an active metabolite of phenacetin with antipyretic and analgesic effects and has been extensively used as a painkiller. Currently, the problem of pharmaceuticals in water and sewage is common, especially in highly urbanized countries. Laboratory-scale experiments were carried out using an adsorbent—granulated activated carbon (WD-extra)—to remove acetaminophen (ACT) from water. The initial concentration of acetaminophen was 20 mg ACT/dm^3^. The adsorption kinetics, influence of the pH on adsorption and dose of the used adsorbent were determined under batch conditions. The adsorption of ACT on activated carbon was more efficient when the water solution was acidic (at pH 2, it was the most effective). The highest percentage of removal (99%) was obtained for the WD-extra dose of 10.0 g/dm^3^. The time taken to establish the dynamic equilibrium of the system was 60 min. The effectiveness of adsorption was determined based on the Freundlich and Langmuir adsorption isotherms. It was found that WD-extra activated carbon effectively removed ACT from water solutions.

## 1. Introduction

The first information about the presence of pharmaceuticals in water appeared in the 1970s in the United States. These were salicylic acid and caffeine [1]. In Poland, the first data on the occurrence of drugs in the environment were obtained in 2001 [2]. A very large group of consumed drugs are non-steroidal anti-inflammatory drugs and analgesics. Nowadays, ibuprofen and acetaminophen are two of the most commonly consumed medicines in all countries in the world. Acetaminophen has been widely used not only as an analgesic and antipyretic but also as a main ingredient in anti-influenza drugs around the world. Moreover, it is an over-the-counter pharmaceutical [3]. ACT is not completely metabolized in the human body; so after ingestion, it is excreted (with urine and feces in both native and metabolized forms) and finally may arrive at sewage systems, wastewater treatment plants and water bodies, thus having a significant negative impact on water systems [4]. Moreover, acetaminophen is toxic and poses a potential threat to living organisms, both animals and humans. In addition to its pharmaceutical applications, ACT may be consumed through drinking water. Its presence in the human body may cause liver and kidney damage, genotoxicity and the disruption of hormone production [5]. It has often been detected in sewage in various countries in Asia, Europe and America. The most common sources of acetaminophen are hospitals, sewage treatment plants, households, veterinary medicine and the pharmaceutical industry [6]. Another source of pollution in wastewater is the improper disposal of expired drugs, which most often end up in toilets or are thrown into the garbage [7]. Pharmaceuticals can also be transported to water systems by infiltration or surface runoff. Cemeteries may also be a source of a small amount of pharmaceuticals [6]. All of the sources mentioned above indicate that the removal of these pharmaceuticals from aqueous solutions is a very serious and increasingly prevalent problem, especially with regard to the preservation of environmental quality [2,8]. Commonly used conventional wastewater treatment technologies do not guarantee the complete removal of contaminants. In order to effectively eliminate ACT (and other organic pollutants) from water, many studies have been carried out, and various technological systems have been evaluated, at both a laboratory and pilot scale. Biological processes, advanced oxidation processes (photodegradation), membrane processes and adsorption using various adsorbents have been taken into account [9,10,11,12]. The decontamination of ACT can also be achieved through the biosorption process [13]. In view of the fact that the mechanisms of action of the mentioned processes are different, the principles of operation for various technological systems also differ; thus, the removal of ACT from aqueous solutions occurs with different levels of efficiency. Such factors may include the ACT concentration, source and geographical and environmental conditions [4]. Nevertheless, technological systems based on the mentioned individual processes require highly technological regimes and are relatively expensive. Granulated activated carbon (GAC) has a very wide and high-yield range of applications in water treatment processes. A carbon bed can act as an adsorption filter in an entire technological system; it can also act as a filter protecting the water treatment process against sudden fluctuations in the quality of the collected water, or as an adsorbent included in the technological process in the event of incidental unforeseen accidents. Apart from its sorption properties, granulated carbon is characterized by favorable physical parameters such as abrasion resistance, a specific density and favorable hydraulic properties. Therefore, the use of GAC in water treatment plants has a very diverse scope depending on the parameters of the carbon used as well as the individual properties of water. The use of granulated activated carbon in ACT adsorption from water may prove to be a simple and economically beneficial method [14,15]. 

The novelty and aims of this research were to determine ACT adsorption efficiency from aqueous solutions on commercially available granulated activated carbon WD-extra, purchased from the Polish company, GRYF-SKAND, in terms of its use for application purposes, and to establish how the adsorption process was influenced by various factors such as contact time, solution pH and a variable dose of WD-extra.

## 2. Materials and Methods

### 2.1. Used Materials

Activated carbon WD-extra (granulated), manufactured by GRYF-SKAND from Hajnowka in Poland, was applied as an ACT adsorbent. The WD-extra carbon used was hard bituminous coal and had a relatively large specific surface, which suggests the coal had a well-developed porous structure. 

Before determining the specific surface area, raw (commercial) activated carbon was initially prepared. A total of 300 g of WD-extra activated carbon was poured into a 1000 cm^3^ beaker. Beforehand, the dusty fraction was removed by rinsing it several times with distilled water. Then, the carbon was dried in a dryer at 105 °C for 24 h. 

The specific surface area of this commercially available WD-extra was 1020 m^2^/g. Its specific surface area was determined by the BET method for N_2_ adsorption at 76 K in liquid nitrogen using a Sorpty 1750 BET (Carlo Erba analyzer, Milan, Italy). The most important parameters characterizing the studied activated carbon are presented in Table 1 (the data were provided by the WD-extra manufacturer).

A model solution with an initial adsorbate ACT concentration of 20 mg/dm^3^ was prepared by dilution in deionized water (0.065 µS cm^−1^, SolPure-7 water purification systems, from Poll-Lab (Bielsko-Biala, Poland). The pH of the model solution ranged from 6.9 to 7.0. It was established by the potentiometric method using Mettler Toledo Five Easy Plus pH meter (FEP20, Zürich, Switzerland). In order to determine the equilibrium and adsorption kinetics, the experiments were conducted under non-flow conditions combined with stirring (40 rpm) in an orbital laboratory shaker (WL-2000 JW Elektronic, Warsaw, Poland). 

Determination of the ACT concentration was performed indirectly by measuring the absorbance at a wavelength of λ = 254 nm using a spectrophotometer (Shimadzu UV-1601, Kyoto, Japan) with quartz cuvettes with a 1 cm-thick absorption layer. For this purpose, correlation standard curves were created between ACT (mg/dm^3^) and UV absorbance. For more precise measurements, two standards curves were determined for different concentration ranges. The first curve was in the range of lower concentrations from 0 to 5 mg/dm^3^, and the other in the range of higher concentrations from 5 to 25 mg/dm^3^. All of the chemicals used were of analytical grade.

### 2.2. Used Methods

#### 2.2.1. Adsorption Kinetics Studies

The experiment was carried out as follows: using a laboratory scale (±0.1 mg, Discovery Analytical DV314CM, OHAUS, Parsippany, NJ, USA) a doses of 1 g WD-extra adsorbent were added to 9 beakers containing 250 cm^3^ of the ACT model solution at a concentration of 1 g/dm^3^ and shaken for various times. The time range was from 5 to 180 min. Next, the samples were subjected to sedimentation for 24 h. Then, the liquid phase was filtered for each sample using syringe cellulose filters (0.2 μm MCE, Whatman, Maidstone, UK) and the ACT concentration was determined. 

**Figure 1:**
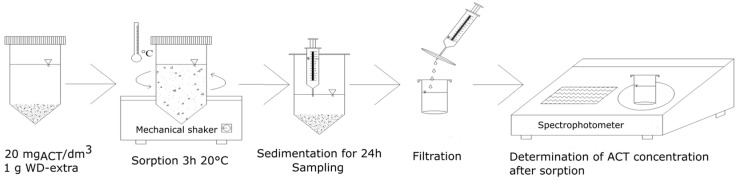
The subsequent stages of the conducted experiments.

After setting up shaking times (adsorbent–adsorbate contact), the ACT content (C_e_) was determined in the solutions. The concentration of ACT in the solid phase (q_e_) was calculated from the balance equation (Equation (1)). The subsequent stages of the conducted experiments are shown schematically in Figure 1.

Empirical equations such as the pseudo-second-order (PSO) and pseudo-first-order equations (PFO) originally given by Lagergren [16] are usually used to describe the kinetic processes of adsorption at the solution–solid interface.

In this case, the modeling process consisted of fitting the measured experimental data with two equations, PFO (Equation (2)) and PSO (Equation (4)), and selecting the one that best correlated with the data. It is better to correlate experimental data with equations in linear form, so equations in differential form were transformed to linear equations (Equations (3) and (5)), respectively.

The adsorption capacity in a state of equilibrium was determined from the equation:

(1) $$q_{e}=V\cdot \left(C_{0}-C_{e})/(m\right)$$

where:

q_e_—Equilibrium adsorption capacity of ACT in the adsorbent—solid phase [mg/g];
V—Volume of adsorptive ACT solution [dm^3^];
C_0_—Initial adsorbate ACT concentration in liquid phase [mg/dm^3^];
C_e_—Equilibrium adsorbate concentrations in the solution [mg/dm^3^];
m—Mass of adsorbent used [g].

The differential form of the PFO model equation is as follows:

(2) $$\frac{dq(t)}{dt}=k_{1}(q_{ekin}-q\left(t\right))$$

where:

t—contact time [h];
q—the amount of adsorbate (depend on time);
q_ekin_—the value of q in dynamic equilibrium, i.e., q (t → ∞) = q_e_;
k_1_—kinetic constant [1/h].

The linear form of models for data analysis:

(3) $$\ln(q_{ekin}-q(t))=\ln q_{ekin}-k_{1}t$$

The differential form of the PSO model equation is as follows:

(4) $$\frac{dq(t)}{dt}=k_{2}{\left(q_{ekin}-q\left(t\right)\right)}^{2}$$

where:

k_2_—kinetic constant [mg/g·h].

The linear representation with the constant k_2_ and q_e_ is:

(5) $$\frac{t}{q(t)}=\frac{1}{k_{2}q_{ekin}^{2}}+\frac{t}{q}$$

#### 2.2.2. The Influence of pH on the Adsorption Process

One of the parameters controlling the adsorption process of weak electrolytes (e.g., acetaminophen) on adsorbents (including activated carbon) is the pH of the adsorptive medium which affects the electrostatic interactions between the adsorbents and the adsorbates.

Batch experiments were performed at pH ranges from 2 to 10. The pH of the solution was adjusted using the reagents 0.1 mol/dm^3^ HCl and 0.1 mol/dm^3^ NaOH. A glass vessel containing 250 cm^3^ of ACT model solution and 0.25 g of WD-extra was shaken for 1 h at room temperature, sedimentation was carried out for 24 h and then the adsorbent was filtered using syringe cellulose filters. Then, the residual concentration of ACT in the filtrate was evaluated.

#### 2.2.3. The Influence of the WD-Extra Doses on the Adsorption Effectiveness

As analogously described above, the experiments were performed under batch conditions for 9 samples using glass vessels with 250 cm^3^ of the model solution of ACT. Increasing doses of an adsorbent were added to each sample. All samples were protected by insulating material and shaken for 1 h at room temperature. The control of ACT concentration was carried after 24 h of sedimentation.

#### 2.2.4. Adsorption Equilibrium Studies

The dependence of the amount of adsorbed ACT on its equilibrium concentration room temperature of 20 °C is presented in the form of adsorption isotherms. Two physical adsorption models were analyzed corresponding to the following equations:

(6) Freundlich (F)   qeF=KFCe1nF

(7) $$\mathrm{Langmuir}\;(L)\;\;\;\;\;q_{eL}=q_{m}K_{L}C_{e}/\left(1+KC_{e}\right)$$

where:

K—adsorption equilibrium constant, related to the sorption capacity of the material, and the subscripts K_F_, K_L_ correspond to the isotherm name, respectively [dm^3^/mg];
n—isotherm constant determining heterogeneity of sorbent surface (dimensionless);
q_e_—equilibrium adsorption capacity [mg/g];
q_m_—maximum adsorption capacity [mg/g];
C_e_—equilibrium concentration [mg/dm^3^].

## 3. Results of Studies and Discussion

### 3.1. The Kinetics of the Adsorption Process

**Figure 2:**
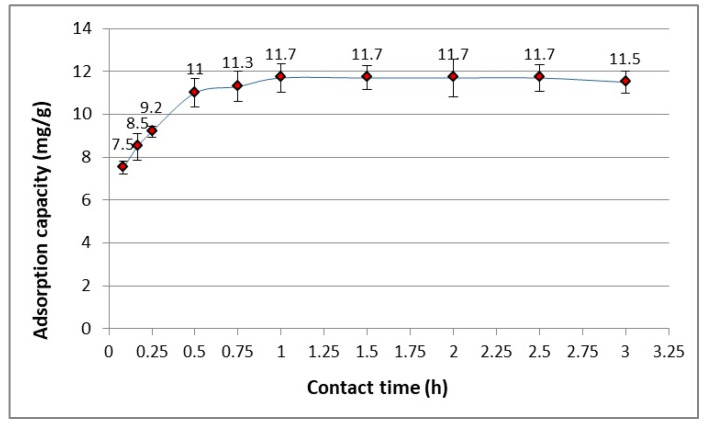
The contact time effect on ACT adsorption onto WD-extra (for C_0_ = 20 mg/dm^2^, 250 cm^3^ of ACT model solution and 0.25 g of WD-extra).

The mechanism of mass exchange in adsorption is a diffusive and very complex process. It is specified by phases such as convection in the adsorbate solution, diffusion of the adsorbent to the interfacial surface, diffusion through the interfacial surface and in the liquid phase inside the pores. The last stage is proper adsorption, i.e., reactions at the active sites of the adsorbate. Therefore, the adsorption process occurs rapidly in the first stage, i.e., in the first minutes. This is due to the large number of available active sites. Despite the interaction (physical or chemical) of the active sites being the fastest, the adsorption kinetics is limited by these slower diffusion processes, which mainly take place under non-flow conditions. This phenomena occurs at the phase boundary and inside the pores of the adsorbent. Slower adsorption at a later time may result from the saturation of the active sites. Adsorption in diluted solutions is influenced by shaking time and stirring speed (rpm). The effect of contact time (shaking) on the final adsorbate concentration (ACT) at constant adsorbent doses is illustrated in Figure 2. Based on the conducted studies, it can be observed that the adsorption of ACT was the fastest within the first 5 min. The ACT reduction was 38%, and after another 25 min reached 55%. The ACT concentration close to the adsorption equilibrium (taken as the equilibrium) was obtained after 1 h and ACT concentration decreased up to 58.5%.

**Figure 3:**
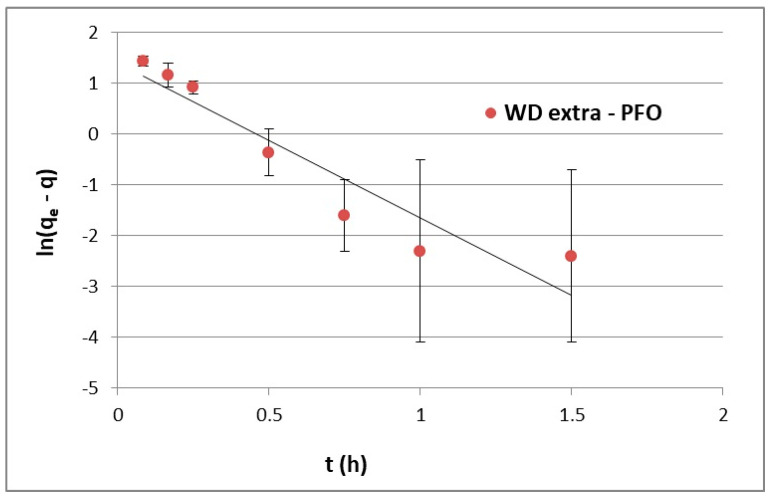
Time profile of acetaminophen adsorption kinetics onto WD-extra described by the pseudo-first-order model.

**Figure 4:**
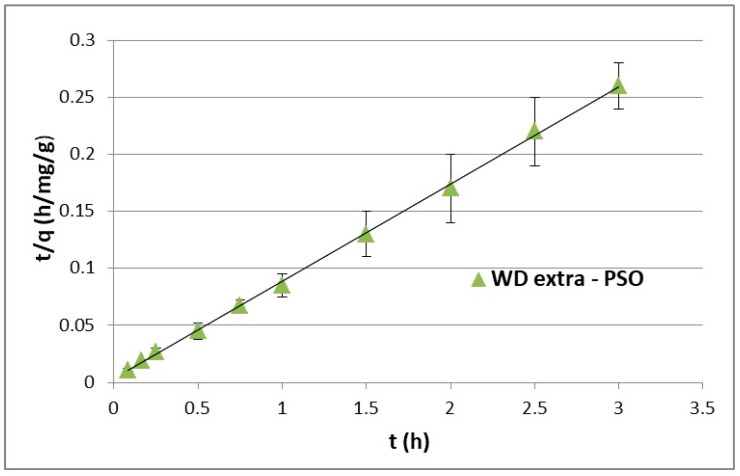
Time profile of acetaminophen adsorption kinetics onto WD-extra described by the pseudo-second-order model.

The adsorption kinetics modeling process explains how the recorded experimental results are correlated with the selected kinetics equations (Equations (2) and (4)). The equation that better describes the data was selected for further consideration, although the selected kinetic model does not explain all of the mechanisms related to the time until maximum sorption capacity is achieved in the system [17]. The fitted kinetic model can only be helpful in determining the factors limiting the process rate (e.g., pH, temperature, etc.). However, Plazinski et al. [17] showed that the PFO and PSO equations similarly describe the adsorption kinetics for a given process with several variable parameters, including temperature and the initial concentration of the adsorbate and do not show qualitative differences in data fitting. The PFO kinetic curve for the WD-extra adsorbent determined as a function of f(t) = ln(q_ekin_ – q(t)) is shown in Figure 3, while pseudo-second order (PSO) plots were prepared as a function of f(t) = t/q_ekin_(t) and illustrated in Figure 4. Calculated constants adsorption rates and the coefficient of determination (R^2^) are presented in Table 2. The rate constants k_1_ (1/h) and k_2_ (mg/g·h) were calculated from the slope coefficients of the determined kinetic lines.

In relation to the experiments and calculations performed, the adsorption kinetics of ACT on WD-extra was consistent with the PSO model, for which the coefficient of determination R^2^ was higher than 0.999. This proves that the adsorption rate increased exponentially in relation to the adsorbate concentration. Even still, the PSO equation better described the adsorption kinetics, and it was assumed that chemisorption largely controlled the process.

### 3.2. The Influence of pH Initial ACT Solution

**Figure 5:**
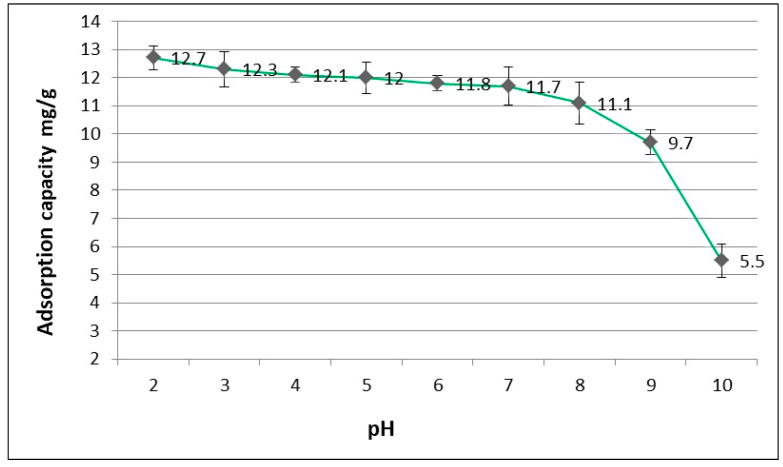
The influence of pH on the ACT adsorption capacity with constant doses of WD-extra.

The effectiveness of ACT adsorption on WD-extra activated carbon depended on the initial pH of the adsorptive. WD-extra carbon has oxygen functional groups that give the carbon surface a polar character. The pH of the solution can additionally influence the positive or negative charge of the surface, which promotes or limits the adsorption process. The concentration of surface functional groups containing oxygen for WD-extra are phenolic groups 0.62 [mmol/g], carbonyl groups 1.12 [mmol/g], total acid groups 1.74 [mmol/g] and the sum of basic groups 2.56 [mmol/g] [18]. It was observed that the adsorption efficiency decreased when the pH of the adsorptive increased. The influence of pH on the amount of adsorbed ACT for constant doses of WD-extra is shown in Figure 5. ACT adsorption on WD-extra was more effective in the pH range 2 to 7. The result can be explained as interactions between the adsorbent and the adsorbate. Acetaminophen is a weak organic acid and the dissociation constant for this compound (pKa) is 9.5. The pH value of the adsorbate is also influenced by the dissociation of the compound. If the pH of the adsorptive is higher than the pKa of the adsorbate, the electrolyte is largely in a dissociated form. Additionally, at a higher solution pH, the surface charge of carbon may be positive. As the pH of the adsorptive decreases (pH < pKa), the more of the adsorbate appears in a non-ionic undissociated form, which may reduce the electrostatic interactions between the adsorbate (ACT) and the adsorbent (WD-extra) and has a positive effect on the adsorption process. The highest ACT adsorption capacity (12.7 mg/g for WD-extra dose of 1 g) was recorded in an acidic environment of pH 2.

### 3.3. The Influence of the WD-Extra Dose on ACT Adsorption

**Figure 6:**
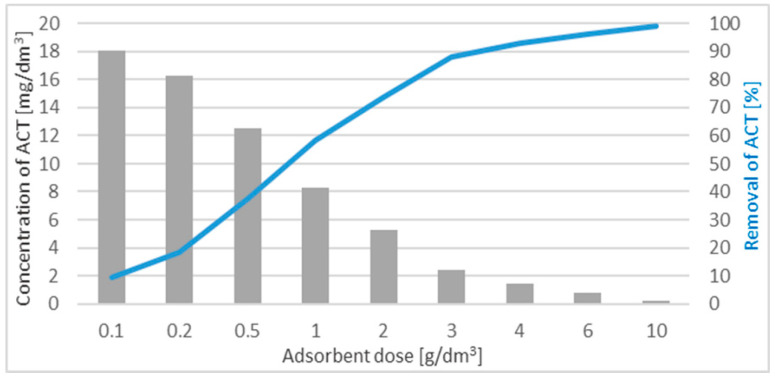
The effect of increasing doses of the adsorbent used for the removal of ACT from the adsorptive.

The most favorable adsorbent dose for effective removal of adsorbate will depend on several parameters, such as the initial concentration of the adsorbate, type of adsorbent, pH, contact time and method of carrying out the process. Therefore, the dose should be determined each time for the assumed factors. For the dose of the adsorbent sample, in the limited range, the net adsorbed amount (mg) is directly proportional to the dose (weight) of the adsorbent sample. The effect of increasing the doses of adsorbent used for the removal of ACT from the adsorptive is shown in Figure 6.

The highest effectiveness (percentage ACT reduction) was obtained for a dose of 10 g/dm^3^ and reached 99%.

### 3.4. Determination of Isotherm Parameters

**Figure 7:**
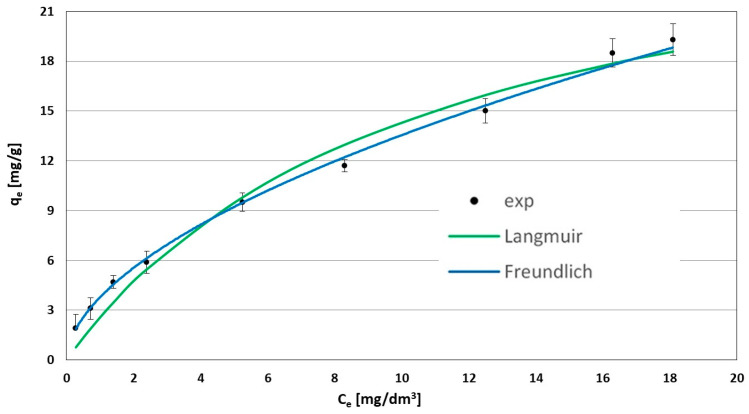
Adsorption isotherms of acetaminophen on WD-extra fitted to the L, F and L-F isotherm equation.

In order to determine the sorption isotherms, the experiments were performed and two adsorption isotherms (Langmuir (L) and Freundlich (F)) were elaborated based on the obtained results. Based on the mathematical equations of the isotherms, the equilibrium constant (K), the adsorption capacity (q_m_) and the constant determining the heterogeneity of the sorbent surface (n) were concluded. The adsorption isotherm constants for WD-extra are presented in Table 3. Adsorption curves are presented as a function of the amount of adsorbed ACT on the adsorbent to the ACT concentration (at adsorption equilibrium), as shown in Figure 7. The modeling of the experimental data using the original form of the nonlinear equations maintains the integrity of the data. This can be achieved by the avoidance of bias in the use of the linearized form of the equation which is based on an operation on data that have already been transformed, leading to errors. Nonlinear expressions of two and three parameters’ adsorption isotherms were used to model the data [19,20]. Nonlinear adsorption model fitting of experimental data was calculated using mathematics-based software Maple 16.2 (Maplesoft) by means of a nonlinear regression method based on the Levenberg−Marquardt algorithm applied to minimize the sum of the squares of the error (SSE) function (Equation (8)):

(8) $$\sum_{i=1}^{N}{\left(q_{e}-q_{emod}\right)}_{i}^{2}=min$$

where:

N—number of experimental points;
q_e_—equilibrium concentration of ACT in the sorbent (solid phase), (mg/g);
q_e mod_—equilibrium concentration of ACT in the sorbent calculated from the model, (mg/g).

Moreover, the quality of the isotherm fit to the experimental data were evaluated based on error functions: mean error (ME); approximation of the standard deviation (σ) of determination (R^2^).

The recorded experimental data correlated well with the three isotherm equations analyzed. The obtained data indicate that the adsorption of ACT on WD-extra occurs including both physical adsorption and chemical adsorption. In essence, the ACT contaminant–carbon surface interactions occur through intermolecular Van der Waal forces and induced dipole interactions and chemical reactions related to the saturation of active sites on the adsorbent materialize between the adsorbate and the adsorbent. Adsorption decreased as the adsorbent coverage increased and tended to achieve the highest efficiency. The good fit of the Freundlich model (Equation (6)) was evidenced by both low values of the standard deviation σ = 0.36 (Equation (9)) and the specific mean error ME = 3.72 (Equation (10)). The R-squared coefficient of determination (Equation (11)) as a measure of the goodness of fit of the model to the experimental values indicated that the fit of all adsorption models used were very good; however, the Freundlich isotherm (R^2^ = 0.999) was practically consistent with the experimental conditions. This confirms that the adsorption mechanism did not include a single-layer adsorption limit but according to the Freundlich isotherm assumptions indicates a multilayer filling of adsorbent by adsorbate molecules. The value of the maximum adsorption achievable capacity for the Langmuir (Equation (7)) R^2^ = 0.977 was 29.3 mg/g resulting from mathematical modelling.

(9) $$\sigma =\sqrt{\frac{1}{N-l}\sum_{i=1}^{N}{\left(q_{e,i}-q_{m,i}\right)}^{2}}$$

(10) $$ME=\frac{1}{N}\sum_{i=1}^{N}\left|\frac{q_{e,i}-q_{m,i}}{q_{e,i}}\right|$$

(11) $$R^{2}=\frac{\sum_{i=1}^{N}{\left(q_{m,i}-\frac{1}{N}\sum_{i=1}^{N}q_{e,i}\right)}^{2}}{\sum_{i=1}^{N}{\left(q_{e,i}-\frac{1}{N}\sum_{i=1}^{N}q_{e,i}\right)}^{2}}$$

For comparison, Nourmoradi et al. [5] conducted a study for the removal of ACT onto modified activated carbon from the Quercus Brantii (oak) acorn for an initial concentration of 200 mg/dm^3^. The results showed that active carbon (modified by KOH) had the highest value of sorption capacity for ACT equal to 45.45 mg/g at pH 3. The findings of the isotherms and kinetics study also showed that the PSO kinetics and Freundlich isotherm models fitted the data much better than others. However, we should bear in mind that the efficiency of adsorption (large adsorption capacity of the adsorbent) depends on many factors; therefore, the comparison of the results obtained with information from the literature reviews has only the orientation character, since the process conditions (initial parameters) in each study differ.

Further research directions should be focused on the use of WD-extra in the filtration process (column adsorption) which may increase its adsorption capacity due to the fact adsorption is carried out in the dynamic conditions and can be comparative to the process carried out in the non-flow conditions, where the additional adsorbent may be regularly added in portions. That is called step adsorption, and therefore a better efficiency of the adsorption process can be achieved; thus, it may indicate possible applications of the research. For example, the removal of naphthalene (also an organic compound similar to ACT) in column adsorption (filtration process) on an activated carbon bed (DTO-carbon) increased the adsorption capacity of the carbon three times in relation to static conditions [21]. Moreover, Ho and Newcombe [22] conducted research where they showed that the biodegradation of organic compounds occurred (next to the adsorption process) during a longer filtration period on activated carbon. This may increase the adsorption efficiency. Moreover, when the adsorption capacity is exhausted, activated carbon can be thermally regenerated and reused.

## 4. Conclusions

This work demonstrated experiments conducted in non-flow conditions and showed that WD-extra activated carbon is a highly effective ACT adsorbent. The total adsorption equilibrium was determined within 1 h. During the first 30 min of adsorption, the process was the most intense. The observed changes in the kinetic parameters in the experimental data were depicted by the pseudo-second-order model (PSO). The adsorption of ACT on activated carbon WD-extra occurred best within pH 2–7. The interpretation of the constant values (K, n) and R^2^ of adsorption isotherms showed that the best model describing the experimental data was the Freundlich isotherm. According to the Freundlich isotherm assumptions, multilayer adsorption (filling of adsorbent by adsorbate molecules) on heterogenous sites takes place. Moreover, the research has shown that the adsorption of ACT on WD-extra occurs including both physical adsorption and chemical adsorption. Nearly 100% of ACT removal efficiency was obtained for a dose of adsorbent of 10 g. The maximum adsorption capacity was 29.3 mg/g.

## Figures and Tables

**Table 1:** Parameters of WD-extra activated carbon.

Parameter	Volume	Unit
Bulk density	390–415	[g/dm^3^]
Granulation	1–1.5	[mm]
Volume of pores (total)	0.85–0.95	[cm^3^/g]
Adsorption of iodine	900–1000	[mg/g]
Dechloration capacity	4–5	[cm]
Mechanical durability	90	[%]

**Table 2:** Rate constants and coefficients of correlation for PFO and PSO.

**WD-Extra**	**(PFO) Pseudo-First-Order**		**(PSO) Pseudo-Second-Order**	
	k_1_ [1/h]	R^2^	k_2_ [mg/g·h]	R^2^
	3.0584	0.8901	2.27	0.9993

**Table 3:** Values of constants of isotherms L and F.

Adsorbent	Constants of Isotherms			ME	*σ*	R^2^
WD-Extra	K	n	q_m_			
Freundlich	K_F_ = 3.7763	1.2828	-	3.72	0.36	0.999
Langmuir	K_L_ = 0.096	-	29.3	17.80	1.07	0.977

## Data Availability

Data are contained within the article.
